# The genetics-BIDS extension: Easing the search for genetic data associated with human brain imaging

**DOI:** 10.1093/gigascience/giaa104

**Published:** 2020-10-17

**Authors:** Clara A Moreau, Martineau Jean-Louis, Ross Blair, Christopher J Markiewicz, Jessica A Turner, Vince D Calhoun, Thomas E Nichols, Cyril R Pernet

**Affiliations:** Sainte Justine Research Center, University of Montreal, 3175 Chemin de la Côte-Sainte-Catherine, Montréal, QC H3T 1C5, Canada, Montreal, QC, Canada; Sainte Justine Research Center, University of Montreal, 3175 Chemin de la Côte-Sainte-Catherine, Montréal, QC H3T 1C5, Canada, Montreal, QC, Canada; Centre for Reproducible Neuroscience, Stanford University, 450 Jane Stanford Way, Stanford, CA 94305, CA, USA; Centre for Reproducible Neuroscience, Stanford University, 450 Jane Stanford Way, Stanford, CA 94305, CA, USA; Imaging Genetics and Informatics Lab, Georgia State University, Atlanta, GA 30302, GA, USA; Center for Translational Research in Neuroimaging and Data Science, Georgia State University, Atlanta, GA 30302, GA, USA; Center for Translational Research in Neuroimaging and Data Science, Georgia State University, Atlanta, GA 30302, GA, USA; Oxford Big Data Institute, Li Ka Shing Centre for Health Information and Discovery, Nuffield Department of Population Health, University of Oxford, Old Road Campus OX3 7LF, UK; Centre for Clinical Brain Sciences & Edinburgh Imaging, University of Edinburgh, 49 Little France Crescent, Edinburgh BioQuarter EH16 4SB, UK

**Keywords:** human brain imaging, genomics, transcriptomics, Brain Imaging Data Structure

## Abstract

Metadata are what makes databases searchable. Without them, researchers would have difficulty finding data with features they are interested in. Brain imaging genetics is at the intersection of two disciplines, each with dedicated dictionaries and ontologies facilitating data search and analysis. Here, we present the genetics Brain Imaging Data Structure extension, consisting of metadata files for human brain imaging data to which they are linked, and describe succinctly the genomic and transcriptomic data associated with them, which may be in different databases. This extension will facilitate identifying micro-scale molecular features that are linked to macro-scale imaging repositories, facilitating data aggregation across studies.

## Introduction

**Figure 1:**
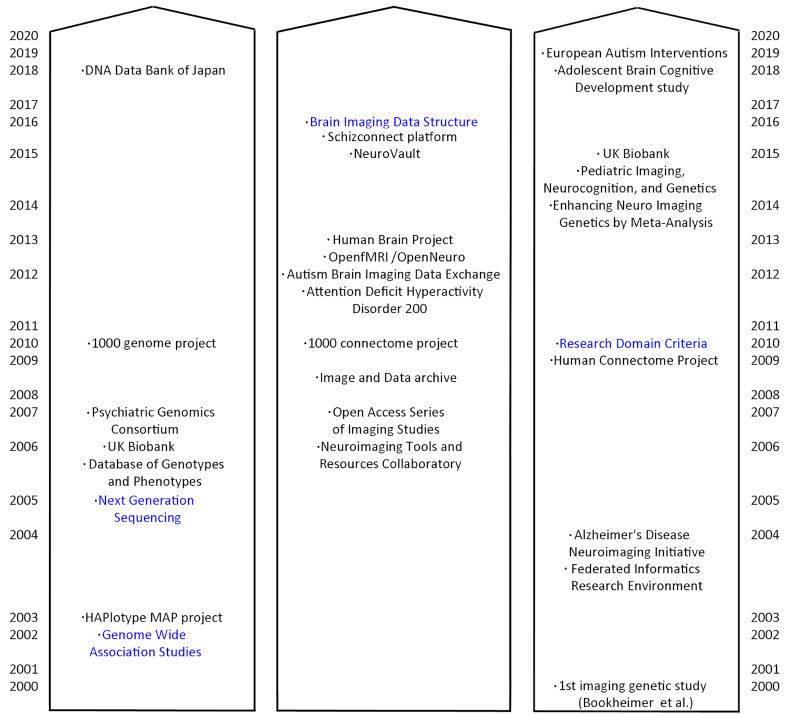
Twenty-first-century view on large-scale projects/databases and key data/metadata tools (in blue) for genetic (left), human brain imaging (middle), and imaging genetic fields (right). First imaging genetic study in 2000 was by Bookheimer et al. [4].

Brain imaging genetics aims at studying the association between brain structure or function and genetic variation [1]. Because gene expression influences cellular mechanisms, which in turn influence neural circuits underlying behaviour, studying associations at the brain level deepens our understanding of gene function at the system level. There is also evidence that using endophenotypes (i.e., brain phenotypes [2, 3]) is better suited to understanding diseases, providing an intermediate description level between genes and clinical phenotypes. While both fields have evolved separately during the 20th century, most recent large-scale efforts combine deep phenotyping with genetic and brain imaging data (Fig. 1).

Both brain imaging and genetics are fields in which researchers are used to sharing data to replicate findings and allow secondary use. Genetic data sharing is however different given the sensitive personal information involved, which must, therefore, be shared through secured and controlled access. Brain imaging, by contrast, is often shared via open data repositories, or authorized access. This has led to different approaches in data sharing for brain imaging genetics: fully secured and controlled for all data (e.g., UK Biobank https://www.ukbiobank.ac.uk/about-biobank-uk/) vs splitting data with open access to brain images but secured access for genetic data (e.g., Human Connectome Project http://www.humanconnectomeproject.org/). The former approach works for large homogenous projects requiring heavy data management while the latter approach is easier, especially for multiple individual smaller studies or multicentric studies with heterogeneous data collection. The Brain Imaging Data Structure (BIDS) describes a way of organizing neuroimaging and behavioural data using dedicated names and dictionaries, documenting metadata [5]. Over time, extensions are being developed and integrated to address users' needs. Here we present the BIDS genetics extension. The primary goal of the BIDS genetics extension is to link BIDS datasets to associated genetic data, especially those existing in separate repositories. The secondary goal is to provide a succinct description of the type of genetic data available, thus enabling searches through multiple imaging datasets.

## The Brain Imaging Data Structure Genetic Descriptor

Data organized according to BIDS have a rigid folder structure and naming convention. Every dataset comes with a dataset_description.json file that contains information relative to authors, funders, ethics, licence, and so forth. To refer to associated genetic data, this file must now include the URL pointing to the genetic data, and optionally the URL of the database, and other associated materials such as dataset descriptor articles. This will make it possible to search quickly through BIDS-compliant repositories for datasets with associated genetic data, possibly allowing automatic downloads provided user credentials are given, because genetic data are usually under controlled access.

Another requirement of BIDS is that a participants.tsv file be included with, at minimum, the subject's identifier. This file can now be used to link the brain imaging and genetic datasets if different pseudo-identifiers are used, making it easy to associate pseudo-IDs without needing to ever access personal information (using participant_id and genetic_id as valid fields). If personal IDs are used, such a file must be provided under secured access only because there is no provision for dealing with this within the extension.

### Extension characteristics and imaging genetic information

**Figure 2:**
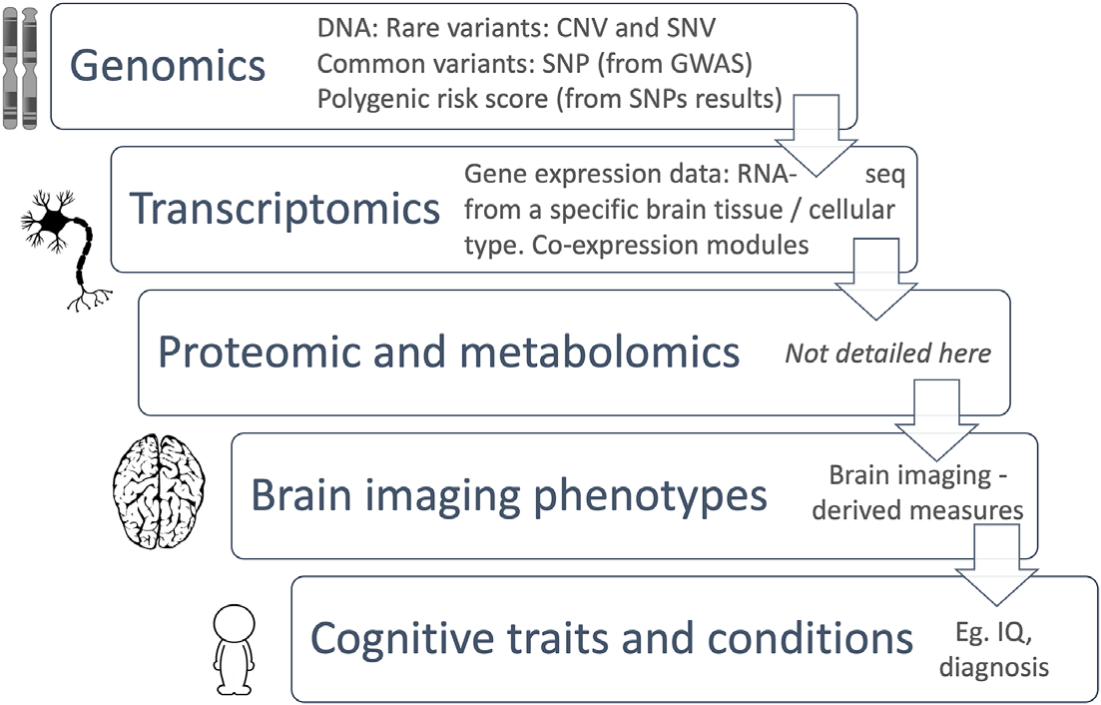
Linking micro- to macro-scale data; CNV: copy number variation; SNV: single-nucleotide variation; SNP: single-nucleotide polymorphism; IQ: intelligence quotient. Icon credit: https://pixabay.com/vectors/brain-human-anatomy-head-1531009/.

This extension of the BIDS project aims to help researchers to structure their molecular (multi-level) and imaging datasets side by side to improve data linkage and search performance. To facilitate metadata search, a genetic_info.json file must be associated with a BIDS dataset describing which type of genetic information is available. Among the multiple available fields, it minimally requires the keys *GeneticLevel*, describing which genetic analyses were carried out, and the *SampleOrigin*. Key values for these fields are “genetic," “genomic," “epigenomic," “transcriptomic," “metabolomic," or “proteomic" [6] (Fig. 2), and “blood," “saliva," “brain," “csf," “breast milk," “bile," “amniotic fluid," or “other biospecimen." If the *SampleOrigin* value is “brain", it is further recommended to add the *TissueOrigin* field (values: “gray matter," “white matter," “csf," “meninges," “macrovascular," or “microvascular"). This can further be refined by indicating the *CellType* field with values taken from the cell ontology [7] and, if the *TissueOrigin* is “gray matter", “white matter", or “csf" (cerebrospinal fluid), using the *BrainLocation* field (values being either MNI coordinates or labels from the Allen Brain Atlas [8]). A last, recommended, field is the *AnalyticApproach*, i.e., the sampling methodology. This is of particular importance because it indicates in greater detail the type of genetic data available using values from the database of Genotypes and Phenotypes (dbGaP - https://www.ncbi.nlm.nih.gov/gap/). As an example, the single-nucleotide polymorphism (SNP) genotyping (Array) and whole-genome sequencing approaches both provide a whole-genome level of genetic information, albeit with some critical differences. SNP genotyping reports genomic data with lower density compared with whole-genome sequencing, which covers more than ∼95% of the genomic DNA. An example of implementation is provided using UK Biobank data at https://github.com/bids-standard/bids-examples/tree/master/genetics_ukbb.

## Conclusion

BIDS is an openly developed, community-led standard to name, document, and organize human brain imaging data, allowing FAIR data sharing [9] and the automation of complex data preprocessing. In just 4 years of existence, it has revolutionized data sharing and analysis in neuroscience, from a wide adoption and a reference for publications to supporting data repository architectures, and it is critical to many open source analysis pipelines. Here, we present the genetic extension that is integrated into the BIDS specification, providing a full documentation of the available fields, along with online examples and a Javascript validator to ensure that datasets are compliant. By adding a genetic descriptor for imaging data, we hope to facilitate data mining to constitute large multi-scale heterogeneous analysis human datasets that reflect human variability, necessary to enhance our understanding of genetic influence on brain phenotypes.

## Availability of Supporting Source Code and Requirements

Project name: Brain Imaging Data Structure

Project home page: https://bids.neuroimaging.io/

BIDS genetics extension: https://bids-specification.readthedocs.io/en/stable/04-modality-specific-files/08-genetic-descriptor.html and https://github.com/bids-standard/bids-specification/blob/master/src/04-modality-specific-files/08-genetic-descriptor.md

Operating system(s): Platform independent

Programming language: Markdown and JavaScript

Other requirements: Node.js to run the validator locally or a web browser to run the validator online

RRID:SCR_016124

License: CC-BY

## Abbreviations

BIDS: Brain Imaging Data Structure; CSF: cerebrospinal fluid; FAIR: findable, accessible, interoperable, and reusable; SNP: single-nucleotide polymorphism.

## Competing Interests

The authors declare that they have no competing interests.

## Authors' Contributions

C.A.M., J.T., V.C., T.E.N., and C.R.P. conceptualized the BIDS extension, and C.A.M. and C.R.P. wrote the manuscript draft; C.A.M., M.J.L., C.M., and C.R.P. wrote the extension and example, and R.B. wrote the javascript validator. All authors contributed to the preparation of the manuscript and/or read and approved the final version.

## Supplementary Material

giaa104_GIGA-D-20-00216_Original_Submission

giaa104_GIGA-D-20-00216_Revision_1

giaa104_Response_to_Reviewer_Comments_Original_Submission

giaa104_Reviewer_1_Report_Original_Submission Jean-Baptiste Poline -- 8/22/2020 Reviewed

## References

[1] Poline J-B, Breeze JL, Frouin V Imaging genetics with fMRI. In: Uludag K, Ugurbil K, Berliner L eds. fMRI: From Nuclear Spins to Brain Functions. New York: Springer;2015:699–738.

[2] John B, Lewis KR Chromosome variability and geographic distribution in insects. Science. 1966;152:711–21. 17797432 10.1126/science.152.3723.711

[3] Gottesman II, Gould TD The endophenotype concept in psychiatry: Etymology and strategic intentions. Am J Psychiatry. 2003;160:636–45. 12668349 10.1176/appi.ajp.160.4.636

[4] Bookheimer SY, Strojwas MH, Cohen MS, et al. Patterns of brain activation in people at risk for Alzheimer's disease. N Engl J Med. 2000;343:450–6. 10944562 10.1056/NEJM200008173430701 PMC2831477

[5] Gorgolewski KJ, Auer T, Calhoun VD, et al. The brain imaging data structure, a format for organizing and describing outputs of neuroimaging experiments. Sci Data. 2016;3:160044. 27326542 10.1038/sdata.2016.44 PMC4978148

[6] Hasin Y, Seldin M, Lusis A Multi-omics approaches to disease. Genome Biol. 2017;18:83. 28476144 10.1186/s13059-017-1215-1 PMC5418815

[7] Malladi VS, Erickson DT, Podduturi NR, et al. The Cell Ontology. Database. 2015;2015, doi:10.1093/database/bav010.

[8] Hawrylycz MJ, Lein ES, Guillozet-Bongaarts AL, et al. An anatomically comprehensive atlas of the adult human brain transcriptome. Nature. 2012;489:391–9. 22996553 10.1038/nature11405 PMC4243026

[9] Wilkinson M, et al. The FAIR Guiding Principles for scientific data management and stewardship. Sci Data. 2016;3:160018. 26978244 10.1038/sdata.2016.18 PMC4792175

