# Supplementary material for: The genetics-BIDS extension: Easing the search for genetic data associated with human brain imaging
Source: Gigascience. 2020 Oct 17;9(10):giaa104. doi: 10.1093/gigascience/giaa104 (PMC7568436; doi:10.1093/gigascience/giaa104)

GigaScience

The genetics-BIDS extension: Easing the search for genetic data associated with human brain imaging

--Manuscript Draft--

**Manuscript Number:**	GIGA-D-20-00216R1
**Full Title:**	The genetics-BIDS extension:<br>Easing the search for genetic data associated with human brain imaging
**Article Type:**	Commentary
**Funding Information:**	
**Abstract:**	Metadata are key in our ability to search databases. Without them, researchers would spend hours examining datasets in the hope to find data with features they are interested in. Brain imaging genetics is at the intersection of two disciplines each one with dedicated dictionaries and ontologies facilitating data search and analysis. Here we present the genetics brain imaging data structure extension: it consists of metadata files for human brain imaging data to which they are linked to and describe succinctly the genomic and transcriptomic data associated to them, possibly in different databases. This extension will facilitate identifying micro-scale molecular features that are linked to macro-scale imaging repositories facilitating data aggregation across studies.
**Corresponding Author:**	Cyril Pernet, PhD<br>The University of Edinburgh<br>Edinburgh, Scotland UNITED KINGDOM
**Corresponding Author Secondary Information:**	
**Corresponding Author's Institution:**	The University of Edinburgh
**Corresponding Author's Secondary Institution:**	
**First Author:**	Cyril Pernet, PhD
**First Author Secondary Information:**	
**Order of Authors:**	Cyril Pernet, PhD
	Clara Moreau, PhD
	Martineau Jean-Louis
	Ross Blair
	Christopher Markiewicz, PhD
	Jessica Turner, PhD
	Vince Calhoun, PhD
	Thomas Nichols, PhD
**Order of Authors Secondary Information:**	
**Response to Reviewers:**	The abstract contains words like "ontologies" and "databases" but the work is about lexicons and disk / filename organization. The abstract should be rewritten to better describe the work.<br><br>Thank you for noticing that. it was indeed not clear that ‘ontologies’ was meant for each discipline and not for imaging genetics; the abstract has been amended to make this clearer and also to indicate that the extension is about metadata files.<br><br>Github repo: while the BIDS overaching project link is given, it takes a while to find where the specific sub field standard is described. It seems that the specific subfield standard is described in page 72 and 73 of the current BIDS specification, but pointing

Powered by Editorial Manager® and ProduXion Manager® from Aries Systems Corporation

	directly to a web page and a git repository containing the documentation would help the reader.<br><br>This was in the hyperlink within the text, but we have now made it more explicit.<br><br>Links to related projects and organization: This short paper does not reference or place the work in the context of standardization organizations in genetic and neuroscience.<br><br>We appreciate that we haven't linked to other efforts in the domain, but after adding/rephrasing according to other comments and the 1200 words limit and maximum of 10 references, we simply do not have the space or opportunity to properly do that - we have however made a new figure showing that new large projects have imaging and genetics - we hope this is acceptable.<br><br>Links to datasets available into the format: The standard is probably instantiated in at least one dataset, and hopefully in several datasets. It would be important for the reader to be able to see explore these datasets.<br><br>Yes we now link to the example from the UK biobank data at: https://github.com/bids-standard/bids-examples/tree/master/genetics_ukbb<br><br>Relation to linked data principles. Since this work attempts to ease linking brain and genetic/genomic data, it would be useful to see how the json file could be turned into a json-ld file (associating a context and pointing to specific definitions for the terms).<br><br>We decided not to discuss this here given space limitation but 100% yes - also there is a similar idea in the BEP28 provenance extension: A feature request on this has been opened at https://github.com/bids-standard/bids-specification/issues/577<br><br>## Specific remarks or questions:<br>- The "URL pointing to the genetic data" : does this mean that "wget URL" will download all data ?<br><br>If users have credentials to the database, yes - added in the manuscript.<br><br>- In which circumstances the link of the genetic participant and the brain imaging data could be sensitive and if it is, how can this be handled ?<br><br>This would be the case if one of the ID is the actual participant name and/or have date of birth. This is left to users to use pseudoIDs. We point to that indicating that if personal IDs are used, this must stay under controlled access.<br><br>- It is unclear which values are permitted in the for the "GeneticLevel" or "SampleOrigin" or "TissueOrigin"<br><br>This section was rephrased to make this clearer.<br><br>- If AnalyticApproach is of particular importance, why is it not a required field ?<br><br>It is a recommended field, but since coverage is not key in all analyses, it was not deemed mandatory.<br><br>- While this is not specific to this work, it would be interesting to see how a json schema could be built for the validation of the specification.<br><br>Maybe another feature request to open on BIDS - not sure if that's not redundant with the circle.ci validation though.
**Additional Information:**	
**Question**	**Response**
Are you submitting this manuscript to a special series or article collection?	No

**Experimental design and statistics**<br><br>Full details of the experimental design and statistical methods used should be given in the Methods section, as detailed in our Minimum Standards Reporting Checklist. Information essential to interpreting the data presented should be made available in the figure legends.<br><br>Have you included all the information requested in your manuscript?	No
If not, please give reasons for any omissions below.<br><br>as follow-up to "**Experimental design and statistics**<br><br>Full details of the experimental design and statistical methods used should be given in the Methods section, as detailed in our Minimum Standards Reporting Checklist. Information essential to interpreting the data presented should be made available in the figure legends.<br><br>Have you included all the information requested in your manuscript?<br><br>"	commentary paper on metadata descriptor
**Resources**<br><br>A description of all resources used, including antibodies, cell lines, animals and software tools, with enough information to allow them to be uniquely identified, should be included in the Methods section. Authors are strongly encouraged to cite Research Resource Identifiers (RRIDs) for antibodies, model organisms and tools, where possible.<br><br>Have you included the information	No

requested as detailed in our Minimum Standards Reporting Checklist?	
If not, please give reasons for any omissions below.<br><br>as follow-up to "**Resources**<br><br>A description of all resources used, including antibodies, cell lines, animals and software tools, with enough information to allow them to be uniquely identified, should be included in the Methods section. Authors are strongly encouraged to cite Research Resource Identifiers (RRIDs) for antibodies, model organisms and tools, where possible.<br><br>Have you included the information requested as detailed in our Minimum Standards Reporting Checklist?<br><br>"	not relevant
**Availability of data and materials**<br><br>All datasets and code on which the conclusions of the paper rely must be either included in your submission or deposited in publicly available repositories (where available and ethically appropriate), referencing such data using a unique identifier in the references and in the "Availability of Data and Materials" section of your manuscript.<br><br>Have you have met the above requirement as detailed in our Minimum Standards Reporting Checklist?	Yes

Manuscript

Click here to access/download;Manuscript;Copy of BIDS genetics.docx

The genetics-BIDS extension:
Easing the search for genetic data associated with human brain imaging

Clara A Moreau[1], Martineau Jean-Louis[1], Ross Blair[2], Chris Markiewicz[2], Jessica Turner[3], Vince Calhoun[4], Thomas E. Nichols[5] & Cyril R Pernet[6]

[1] Sainte Justine Research Center, University of Montreal, Montreal QC, CA
[2] Centre for Reproducible Neuroscience, Stanford University, USA
[3] Imaging Genetics and Informatics Lab, Georgia State University, USA
[4] Center for Translational Research in Neuroimaging and Data Science, Georgia State University, USA
[5] Oxford Big Data Institute, Li Ka Shing Centre for Health Information and Discovery, Nuffield Department of Population Health, University of Oxford, UK
[6] Centre for Clinical Brain Sciences & Edinburgh Imaging, University of Edinburgh, UK

Abstract

Metadata are key in our ability to search databases. Without them, researchers would spend hours examining datasets in the hope to find data with features they are interested in. Brain imaging genetics is at the intersection of two disciplines each one with dedicated dictionaries and ontologies facilitating data search and analysis. Here we present the genetics brain imaging data structure extension: it consists of metadata files for human brain imaging data to which they are linked to and describe succinctly the genomic and transcriptomic data associated to them, possibly in different databases. This extension will facilitate identifying micro-scale molecular features that are linked to macro-scale imaging repositories facilitating data aggregation across studies.

Keywords

Human brain imaging, genomics, transcriptomics, Brain Imaging Data Structure

Introduction

Brain imaging genetics aims at studying the association between brain structure or function and genetic variation [1]. Since gene expression influences cellular mechanisms, which in turn influences neural circuits underlying behaviour, studying associations at the brain level deepens our understanding of gene function at the system level. There is also evidence that using endophenotypes (i.e. brain phenotypes [2, 3]) is better suited to understand diseases, providing an intermediate description level between genes and clinical phenotypes. While both fields have evolved separately during the 20th century, most of recent large-scale efforts combine deep phenotyping with genetic and brain imaging data (figure 1).

[Figure 1]

Figure 1. Twenty-first-century view on large scale projects/databases and key data/meta-data tools (in blue) for genetic (left), human brain imaging (middle) and imaging genetic fields (right).

Both brain imaging and genetics are fields in which researchers are used to share data in order to replicate findings and allow secondary usage. Genetic data sharing is however different given

the sensitive personal information that must, therefore, be shared through secured and controlled access. Brain imaging, by contrast, is often shared via open data repositories, or authorized access. This has led to different approaches in data sharing for brain imaging genetics: fully secured and controlled for all data (e.g. UK biobank [4]) vs. splitting data with open access to brain images but secured access for genetic data (e.g. Human Connectome Project [5]). The former approach works for large homogenous projects requiring heavy data management while the latter approach is easier, especially for multiple individual smaller studies or multicentric studies with heterogeneous data collection. The Brain Imaging Data Structure (BIDS) describes a way of organising neuroimaging and behavioural data using dedicated names and dictionaries, documenting metadata [6]. Over time, extensions are being developed and integrated to address users' needs. Here we present the BIDS genetics extension. The primary goal of the BIDS genetics extension is to link BIDS datasets to associated genetic data, especially those existing in separate repositories. The secondary goal is to provide a succinct description of the type of genetic data available, thus enabling searches through multiple imaging datasets.

The brain imaging data structure genetic descriptor

Data organized according to BIDS have a rigid folder structure and naming convention. Every dataset comes with a *dataset_description.json* file that contains information relative to authors, funders, ethics, licence, etc. To refer to associated genetic data, this file must now include the URL pointing to the genetic data, and optionally the URL of the database, and other associated materials like dataset descriptor articles. This will allow searching quickly through BIDS compliant repositories for datasets with associated genetic data, possibly allowing downloading automatically provided user credentials are given, since genetic data are usually under controlled access.

Another requirement of BIDS is that a *participants.tsv* file is included with, at minimum, the subject’s identifier. This file can now be used to link the brain imaging and genetic datasets if different pseudo-identifiers are used, making it easy to associate pseudo-IDs without needing to ever access personal information (using `participant_id` and `genetic_id` as valid fields). If personal IDs are used, such a file must be provided under secured access only as this is not dealt with, within the extension.

Extension characteristics and imaging genetic information

This extension of the BIDS project aims to help researchers to structure their molecular (multi-level) and imaging datasets side-by-side in order to improve data linkage and search performance. To facilitate metadata search, a *genetic_info.json* file must be associated with a BIDS dataset describing which type of genetic information is available. Among the multiple available fields, it minimally requires the keys `GeneticLevel` describing which genetic analyses were carried out and the `SampleOrigin`. Key values for these fields are *genetic, genomic, epigenomic, transcriptomic, metabolomic or proteomic* [7] (figure 2), and *blood, saliva, brain, csf, breast milk, bile, amniotic fluid, or other biospecimen*. If the `SampleOrigin` value is brain, it is further recommended to add the `TissueOrigin` field (values: *gray matter, white matter, csf, meninges, macrovascular* or *microvascular*). This can further be refined by indicating the `CellType` field with values taken from the cell ontology [8] and, if the `TissueOrigin` is gray matter, white matter or CSF, use the `BrainLocation` field (values being either MNI coordinates or labels from the Allen Brain Atlas [9]). A last, recommended, field is the `AnalyticApproach`, that is the sampling methodology. This is of particular importance since it indicates in greater detail the type of genetic data available using values from the database of Genotypes and

Phenotypes (dbGaP [10]). As an example, the single nucleotide polymorphisms (SNP) genotyping (Array) and whole genome sequencing approaches provide both a whole genome level of genetic information, albeit with some critical differences. The SNP genotyping reports genomic data with lower density compared to the whole genome sequencing which covers over ~95% of the genomic DNA. An example of implementation is provided using UK Biobank data at: https://github.com/bids-standard/bids-examples/tree/master/genetics_ukbb.

Conclusion

BIDS is an openly developed, community-led standard to name, document and organize human brain imaging data, allowing FAIR data sharing and the automation of complex data preprocessing. In just four years of existence, it has revolutionized data sharing and analysis in neuroscience, from a wide-adoption and a reference for publications to supporting data repository architectures, and it is critical to many open-source analysis pipelines. Here, we present the genetic extension which is integrated into the BIDS specification providing a full documentation of the fields that may be provided, along with online examples and a Javascript validator to ensure datasets are compliant. By adding a genetic descriptor for imaging data, we hope to facilitate data mining to constitute large multi-scale heterogeneous analysis human datasets that reflect human variability, necessary to enhance our understanding of genetic influence on brain phenotypes.

[Figure 2]

Figure 2: Linking micro- to macro-scale data. (CNV: copy number variation, SNV: single nucleotide variation, SNP: single nucleotide polymorphisms, PRS: polygenic risk score, eQTL: expression quantitative trait loci, IQ: intelligence quotient, ADHD: attention deficit hyperactivity disorder) - icon credit: https://pixabay.com/vectors/brain-human-anatomy-head-1531009/

Author contributions

CAM, JT, TN, VC and CP conceptualized the BIDS extension, CAM and CP wrote the manuscript draft; CAM, MJL, CM and CP wrote the extension and example, RB wrote the javascript validator. All co-authors have contributed to the preparation of the manuscript, and/or read and approved the final version.

Authors declare no conflict of interest

Availability of supporting source code and requirements

Project name: Brain Imaging Data Structure
Project home page: https://bids.neuroimaging.io/
BIDS genetics extension: https://bids-specification.readthedocs.io/en/stable/04-modality-specific-files/08-genetic-descriptor.html & https://github.com/bids-standard/bids-specification/blob/master/src/04-modality-specific-files/08-genetic-descriptor.md
Operating system(s): Platform independent
Programming language: Markdown and JavaScript
Other requirements: Node.js to run the validator locally or a web browser to run the validator online
RRID: SCR_016124
License: CC-BY

Acknowledgements

Thank you to Elizabeth Dupre and Stefan Appelhoff for helping to structure and correct the BIDS markdown extension.

References

1. Poline, J-B., Breeze, J.L., Frouin, V. (2015) Imaging Genetics with fMRI. In: Uludag K, Ugurbil, K., Berliner, L. (eds) fMRI: From Nuclear Spins to Brain Functions. Springer, New-York, pp 699–738
2. John B, Lewis KR (1966) Chromosome Variability and Geographic Distribution in Insects. Science 152:711–721. https://doi.org/10.1126/science.152.3723.711
3. Gottesman II, Gould TD (2003) The Endophenotype Concept in Psychiatry: Etymology and Strategic Intentions. AJP 160:636–645. https://doi.org/10.1176/appi.ajp.160.4.636
4. UK Biobank. https://www.ukbiobank.ac.uk/about-biobank-uk/. Accessed 14 Jul 2020
5. Human Connectome Project | Mapping the human brain connectivity. http://www.humanconnectomeproject.org/. Accessed 14 Jul 2020
6. Gorgolewski KJ, Auer T, Calhoun VD, et al (2016) The brain imaging data structure, a format for organizing and describing outputs of neuroimaging experiments. Scientific Data 3:160044. https://doi.org/10.1038/sdata.2016.44
7. Hasin Y, Seldin M, Lusis A (2017) Multi-omics approaches to disease. Genome Biology 18:83. https://doi.org/10.1186/s13059-017-1215-1
8. Malladi VS, Erickson DT, Podduturi NR, et al (2015) The Cell Ontology. Database 2015:. https://doi.org/10.1093/database/bav010
9. Hawrylycz MJ, Lein ES, Guillozet-Bongaarts AL, et al (2012) An anatomically comprehensive atlas of the adult human brain transcriptome. Nature 489:391–399. https://doi.org/10.1038/nature11405
10. Database of Genotypes and Phenotypes (dbGaP). https://www.ncbi.nlm.nih.gov/gap/. Accessed 14 Jul 2020

Figure1

Click here to access/download;Figure;Figure_1.tif

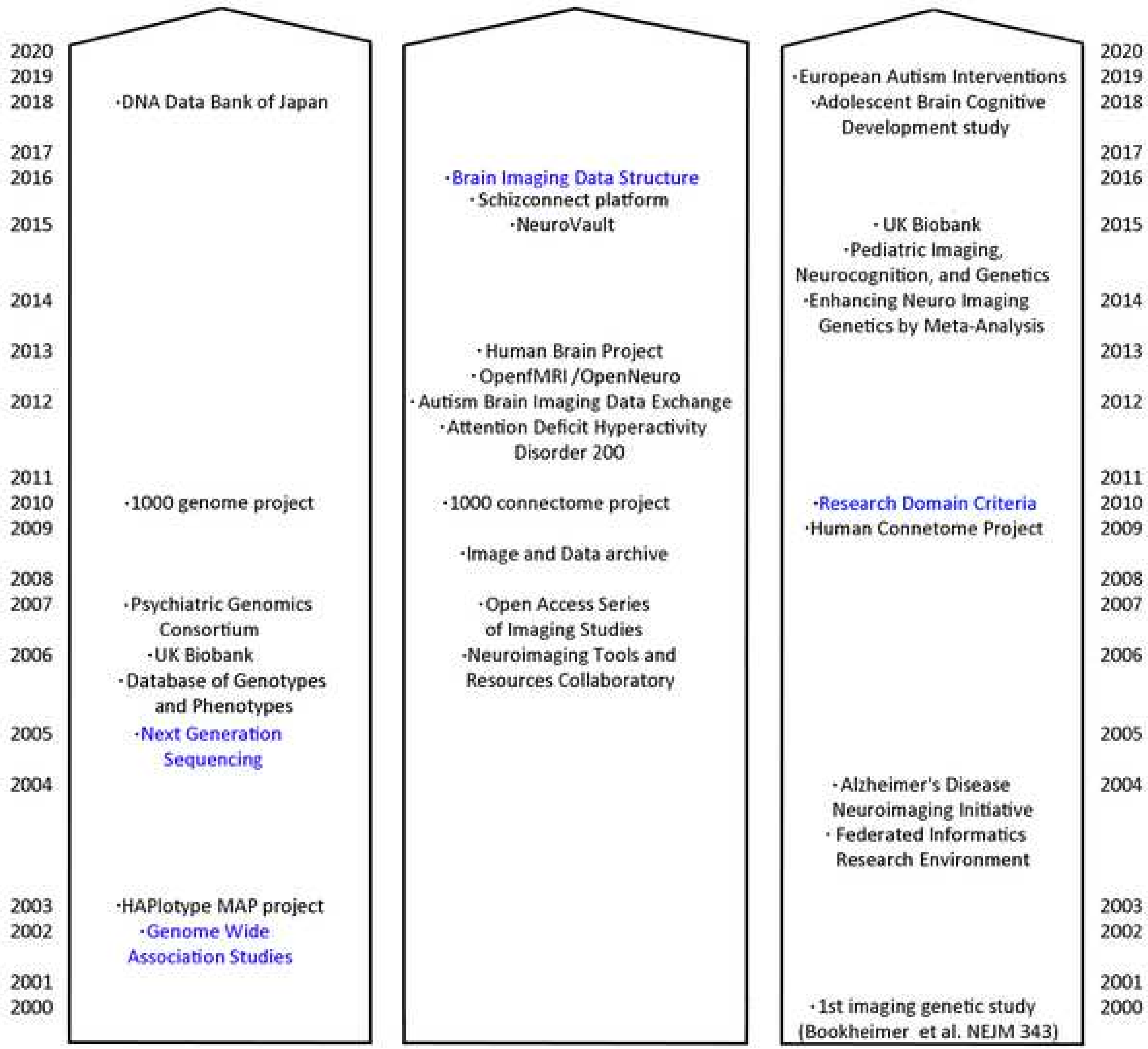

Figure2

Click here to access/download;Figure;Figure_2.tiff

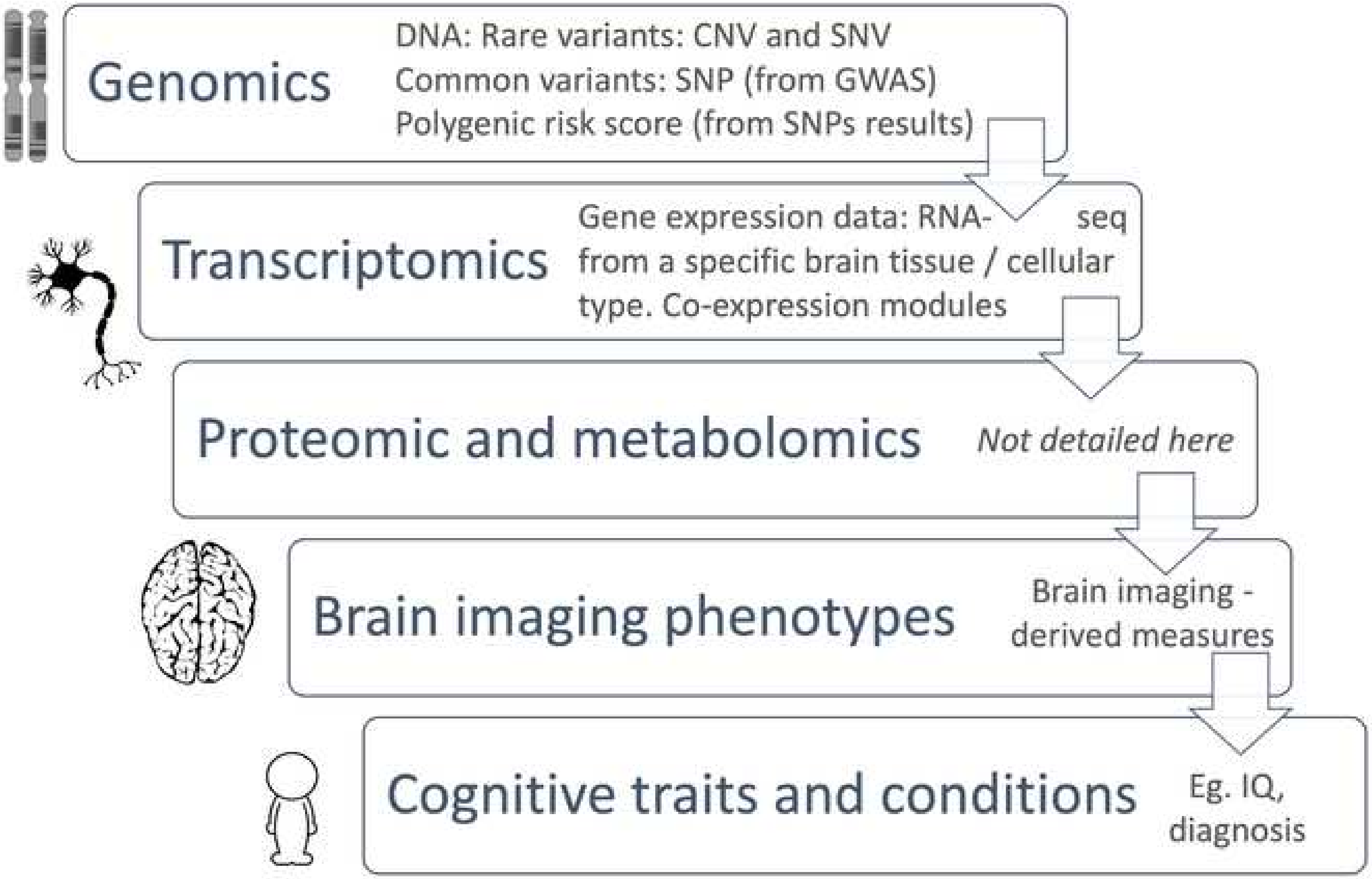

Supplement: giaa104_GIGA-D-20-00216_Revision_1 [file giaa104_giga-d-20-00216_revision_1.pdf]
