# Supplementary material for: The genetics-BIDS extension: Easing the search for genetic data associated with human brain imaging
Source: Gigascience. 2020 Oct 17;9(10):giaa104. doi: 10.1093/gigascience/giaa104 (PMC7568436; doi:10.1093/gigascience/giaa104)
Supplement: giaa104_Response_to_Reviewer_Comments_Original_Submission [file giaa104_response_to_reviewer_comments_original_submission.pdf]

Author's Response To Reviewer Comments

Close

The abstract contains words like "ontologies" and "databases" but the work is about lexicons and disk / filename organization. The abstract should be rewritten to better describe the work.

Thank you for noticing that. it was indeed not clear that ‘ontologies’ was meant for each discipline and not for imaging genetics; the abstract has been amended to make this clearer and also to indicate that the extension is about metadata files.

Github repo: while the BIDS overaching project link is given, it takes a while to find where the specific sub field standard is described. It seems that the specific subfield standard is described in page 72 and 73 of the current BIDS specification, but pointing directly to a web page and a git repository containing the documentation would help the reader.

This was in the hyperlink within the text, but we have now made it more explicit.

Links to related projects and organization: This short paper does not reference or place the work in the context of standardization organizations in genetic and neuroscience.

We appreciate that we haven’t linked to other efforts in the domain, but after adding/rephrasing according to other comments and the 1200 words limit and maximum of 10 references, we simply do not have the space or opportunity to properly do that - we have however made a new figure showing that new large projects have imaging and genetics - we hope this is acceptable.

Links to datasets available into the format: The standard is probably instantiated in at least one dataset, and hopefully in several datasets. It would be important for the reader to be able to see explore these datasets.

Yes we now link to the example from the UK biobank data at: https://github.com/bids-standard/bids-examples/tree/master/genetics_ukbb

Relation to linked data principles. Since this work attempts to ease linking brain and genetic/genomic data, it would be useful to see how the json file could be turned into a json-ld file (associating a context and pointing to specific definitions for the terms).

We decided not to discuss this here given space limitation but 100% yes - also there is a similar idea in the BEP28 provenance extension: A feature request on this has been opened at https://github.com/bids-standard/bids-specification/issues/577

Specific remarks or questions:
- The "URL pointing to the genetic data" : does this mean that "wget URL" will download all data ?

If users have credentials to the database, yes - added in the manuscript.

- In which circumstances the link of the genetic participant and the brain imaging data could be sensitive and if it is, how can this be handled ?

This would be the case if one of the ID is the actual participant name and/or have date of birth. This is left to users to use pseudoIDs. We point to that indicating that if personal IDs are used, this must stay under controlled access.

- It is unclear which values are permitted in the for the "GeneticLevel" or "SampleOrigin" or "TissueOrigin"

This section was rephrased to make this clearer.

- If AnalyticApproach is of particular importance, why is it not a required field ?

It is a recommended field, but since coverage is not key in all analyses, it was not deemed mandatory.

- While this is not specific to this work, it would be interesting to see how a json schema could be built for the validation of the specification.

Maybe another feature request to open on BIDS - not sure if that’s not redundant with the circle.ci validation though.

Close
