# Supplementary material for: The genetics-BIDS extension: Easing the search for genetic data associated with human brain imaging
Source: Gigascience. 2020 Oct 17;9(10):giaa104. doi: 10.1093/gigascience/giaa104 (PMC7568436; doi:10.1093/gigascience/giaa104)
Supplement: giaa104_Reviewer_1_Report_Original_Submission — Jean-Baptiste Poline -- 8/22/2020 Reviewed [file giaa104_reviewer_1_report_original_submission.pdf]

Reviewer Report

Title: The genetics-BIDS extension:Easing the search for genetic data associated with human brain imaging

Version: Original Submission **Date:** 8/22/2020

Reviewer name: Jean-Baptiste Poline

Reviewer Comments to Author:

The genetics-BIDS extension: Easing the search for genetic data associated with human brain imaging
General recommendation
This reviewer finds the work very commendable and thinks that the development of standards is one of the key area to work on to improve the efficiency and solidity of research.
General remarks
The abstract contains words like "ontologies" and "databases" but the work is about lexicons and disk / filename organization. The abstract should be rewriten to better describe the work.
Github repo: while the BIDS overaching project link is given, it takes a while to find where the specific sub field standard is described. It seems that the specific subfield standard is described in page 72 and 73 of the current BIDS specification, but pointing directly to a web page and a git repository containing the documentation would help the reader.
Links to related projects and organization: This short paper does not reference or place the work in the context of standardization organizations in genetic and neuroscience.
Links to datasets available into the format: The standard is probably instanciated in at least one dataset, and hopefully in several datasets. It would be important for the reader to be able to see explore these datasets.
Relation to linked data principles. Since this work attempt to ease linking brain and genetic/genomic data, it would be useful to see how the json file could be turned into a json-ld file (associating a context and pointing to specific definitions for the terms).
Specific remarks or questions:
- The "URL pointing to the genetic data" : does this mean that "wget URL" will download all data ?
- In which circumstances the link of the genetic participant and the brain imaging data could be sensitive and if it is, how can this be handled ?
- It is unclear which values are permitted in the for the "GeneticLevel" or "SampleOrigin" or "TissueOrigin"
- If AnalyticApproach is of particular importance, why is it not a required field ?
- While the keys of the json descriptors are specified,
- While this is not specific to this work, it would be interesting to see how a json schema could be built for the validation of the specification.

Level of Interest

Please indicate how interesting you found the manuscript: Choose an item.

Quality of Written English

Please indicate the quality of language in the manuscript: Choose an item.

Declaration of Competing Interests

Please complete a declaration of competing interests, considering the following questions:

- Have you in the past five years received reimbursements, fees, funding, or salary from an organisation that may in any way gain or lose financially from the publication of this manuscript, either now or in the future?
- Do you hold any stocks or shares in an organisation that may in any way gain or lose financially from the publication of this manuscript, either now or in the future?
- Do you hold or are you currently applying for any patents relating to the content of the manuscript?
- Have you received reimbursements, fees, funding, or salary from an organization that holds or has applied for patents relating to the content of the manuscript?
- Do you have any other financial competing interests?
- Do you have any non-financial competing interests in relation to this paper?

If you can answer no to all of the above, write 'I declare that I have no competing interests' below. If your reply is yes to any, please give details below.

I declare that I have no competing interests

I agree to the open peer review policy of the journal. I understand that my name will be included on my report to the authors and, if the manuscript is accepted for publication, my named report including any attachments I upload will be posted on the website along with the authors' responses. I agree for my report to be made available under an Open Access Creative Commons CC-BY license (http://creativecommons.org/licenses/by/4.0/). I understand that any comments which I do not wish to be included in my named report can be included as confidential comments to the editors, which will not be published.

Choose an item.

To further support our reviewers, we have joined with Publons, where you can gain additional credit to further highlight your hard work (see: https://publons.com/journal/530/gigascience). On publication of this paper, your review will be automatically added to Publons, you can then choose whether or not to claim your Publons credit. I understand this statement.

Yes Choose an item.
